# Assemblages of *Plasmodium* and Related Parasites in Birds with Different Migration Statuses

**DOI:** 10.3390/ijms231810277

**Published:** 2022-09-07

**Authors:** Xi Huang, Zelin Chen, Guocheng Yang, Canwei Xia, Qiujin Luo, Xiang Gao, Lu Dong

**Affiliations:** MOE Key Laboratory for Biodiversity Science and Ecological Engineering, College of Life Sciences, Beijing Normal University, Beijing 100875, China

**Keywords:** haemosporidian, avian migration, parasite assemblage, phylogeny

## Abstract

Migratory birds spend several months in their breeding grounds in sympatry with local resident birds and relatively shorter periods of time at stopover sites. During migration, parasites may be transmitted between migratory and resident birds. However, to what extent they share these parasites remains unclear. In this study, we compared the assemblages of haemosporidian parasites in migratory, resident, and passing birds, as well as the correlations between parasite assemblages and host phylogeny. Compared with passing birds, migratory birds were more likely to share parasites with resident birds. Shared lineages showed significantly higher prevalence rates than other lineages, indicating that common parasites are more likely to spill over from the current host to other birds. For shared lineages, the prevalence was significantly higher in resident birds than in migratory birds, suggesting that migratory birds pick up parasites at their breeding ground. Among the shared lineages, almost two-thirds presented no phylogenetic signal in their prevalence, indicating that parasite transmission among host species is weakly or not correlated with host phylogeny. Moreover, similarities between parasite assemblages are not correlated with either migration status or the phylogeny of hosts. Our results show that the prevalence, rather than host phylogeny, plays a central role in parasite transmission between migratory and resident birds in breeding grounds.

## 1. Introduction

Understanding the associations between parasites and their hosts is of concern when it is correlated with outbreaks of emerging infectious disease [1], especially for parasites that are widely spread or can be transmitted between multiple species. Malaria caused by *Plasmodium* parasites poses a significant threat to public health, and non-human primate malaria strains tend to gradually infect humans, leading to an urgency to study human malaria and its close relatives, among which avian malaria represents an important and understudied strain. During the last century, the transmission of avian *Plasmodium* to Hawaii has caused a sharp population decline in several endemic species [2], indicating the importance of studying the long-distance dispersal of parasites.

Avian *Plasmodium* and related parasites (including *Haemoproteus* and *Leucocytozoon*), also known as avian haemosporidian parasites, are a group of diverse and widespread parasites transmitted by dipteran vectors [3]. They have long been used as a model system for studying host–parasite associations and various factors that may shape these associations [4], with studies mostly focusing on bird hosts due to the difficulty involved in matching parasites to their insect vectors [5]. According to the MalAvi database [6], which compiles the host range and geographical distribution of the published avian haemosporidian parasites, over 4000 lineages have been identified based on molecular methodologies, infecting more than 2000 bird species globally, with the exception of Antarctica. Among the identified lineages, a large number have been detected across multiple continents [7]. Animal migration is considered as an important route for the spreading of parasites and formation of novel host–parasite associations [8]. Approximately 1800 bird species are known to undertake annual migration over varied distances [9], which may mediate the dispersal of those parasite lineages and induce gene flows across different regions [10].

During their annual migration, birds spend several months in their breeding grounds and considerably less time at a few stopover sites. Occasionally, infections in different regions lead to a higher parasite diversity among migratory birds compared to resident birds, which remains roughly the same all year round [11]. However, there is evidence showing that migratory species harbor lower parasite diversity than resident birds because they benefit, through migration, from separation from the infected individual or the avoidance of infected habitats [12], as predicted by the ‘migratory escape’ hypothesis [13]. 

At the same time, parasites carried by migratory birds can be transmitted to resident birds distributed across their migration routine and vice versa. Several studies have shown that cross-species transmission, i.e., the host shift of parasites, plays the main role in shaping the observed host–parasite associations [14,15]. Previous studies on the temporal dynamics of infection patterns have shown that the haemosporidian prevalence in wild birds reaches its peak during the migration and breeding seasons [16], while the infection risk posed to long-distance migratory birds is relatively low at stopover sites [17] and wintering grounds [18]. Therefore, it is tempting to infer that host shifts mostly occur in breeding grounds, but to what extent migratory birds exchange parasites with local resident birds remains unclear. Most studies investigating the host–parasite associations in migration birds have focused on the prevalence or diversity of parasites in a single species, while little is known about the effects of migration on the whole community (however, see [18] for parasite assemblages in wintering grounds).

In order to estimate the assemblages of parasites in relation to bird migration, we hereby investigated the patterns of the host–parasite associations in a natural community in south Beijing, China, during the breeding seasons. In temperate regions, insect vectors are mostly active between May and August [3], coinciding with the breeding period of the bird hosts. During this period, local parasites can be transmitted to birds with different migration statuses, including summer migrants (or migratory birds, breeding in the sampling site), passing birds (passing through the sampling site during migration), and local resident birds. Each year, in our study site, migratory birds stayed for four to six months, while passing birds were normally present for 15 days to one month. If migratory birds are more likely to gain parasites in breeding grounds, we expect that we would detect more parasites shared between resident birds and migratory birds than between resident birds and passing birds. Host shifts of avian haemosporidian often occur among closely related species, as they share important physiological traits and can provide similar environments for parasites [19,20]. In this case, we would expect that migratory birds are more likely to share parasites with resident species which are phylogenetically closely related to them, reflected by similar parasite assemblages in the host species over a short phylogenetic distance. In addition, if birds accumulate infections during their migration pathway, the diversity of parasites would be higher in the migratory birds and passing birds than in the resident birds. If birds benefit from migration by escaping infections, the opposite pattern would be observed.

In this study, we investigated the assemblages of haemosporidian parasites in migratory birds, passing birds, and local resident birds in a natural community, aiming to answer the following questions: (1) to what extent do migratory and passing birds share parasites with local resident species? (2) Are closely related bird species more likely to share the same parasite lineages? (3) Is the parasite assemblage correlated with the phylogeny or migration status of the hosts?

## 2. Results

During 2016–2019, blood samples were collected from 459 individuals belonging to 30 species. The sampled species were divided into three groups based on their migration status, including 13 migratory species, 5 passing species, and 12 resident species (Table S1).

### 2.1. Prevalence and Lineage Diversity of the Haemosporidian Parasites

**Figure 1:**
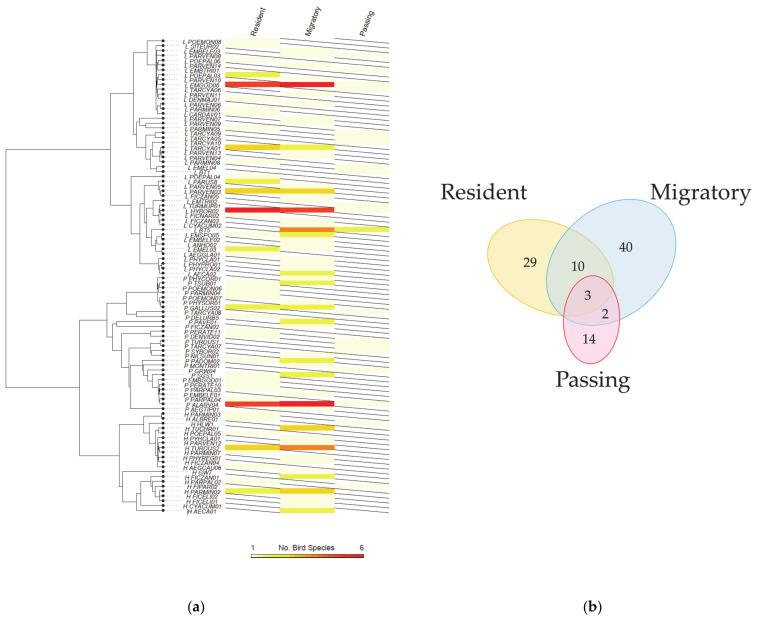
Diversity of haemosporidian lineages detected in this study: (**a**) phylogeny of the lineages and number of host species infected by each represented lineage; (**b**) Venn diagram presenting the numbers of lineages detected in different host groups.

Haemosporidian infections were detected in 204 individuals, representing a general prevalence of 44.4%. The general prevalence among the three host groups showed slight variation (F_2_ = 0.189, *p* = 0.829). A total of 98 distinct lineages were identified, including 21 *Haemoproteus* lineages, 28 *Plasmodium*, and 49 *Leucocytozoon* lineages. Lineages detected in the different host groups were clustered together, without forming any host-specific patterns (Figure 1a). The diversity of the lineages detected in the three host groups showed no significant differences, with or without considering the host phylogeny. However, for the migratory and passing birds, the number of lineages detected in each species was negatively correlated with the average migration distance of the species (*R*^2^ = 0.189, *p* = 0.04), with very low slope (−0.002).

Among the identified lineages, 15 were detected in more than one host group and, therefore, defined as shared lineages (Figure 1b). Of these, ten were detected in both the resident and migratory birds, of which two were *Haemoproteus*, four were *Plasmodium*, and four were *Leucocytozoon*. Two lineages (both *Leucocytozoon*) were detected in the migratory and passing birds, and three (one *Plasmodium* and two *Leucocytozoon*) were detected in all the host groups. Apparently, resident birds share more lineages with migratory birds than with passing birds.

### 2.2. Shared Lineages among the Host Groups

**Figure 2:**
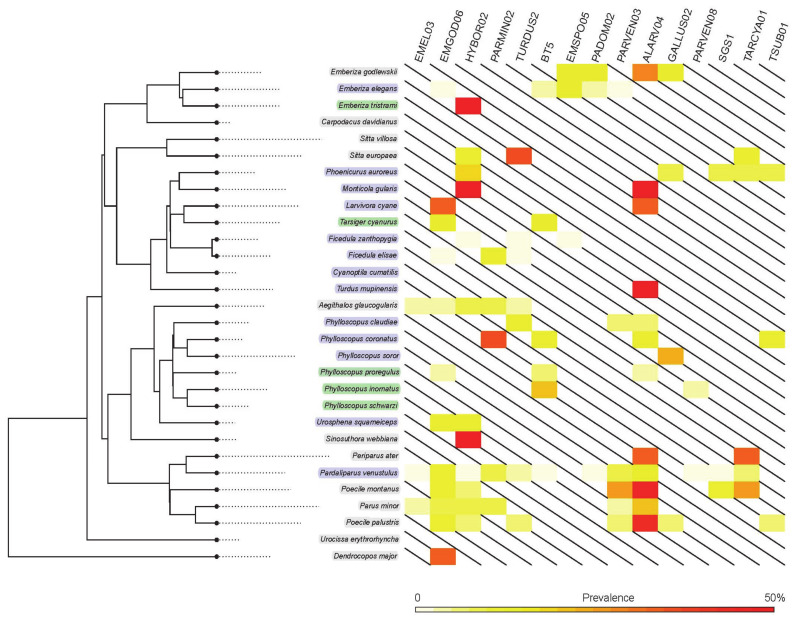
Phylogenetic tree of the bird species collected in this study. Species belonging to three groups are marked in distinct colors (resident: gray, migration: purple, and passing: green). The heatmap on the right panel represents the prevalence of the shared lineages in different host species.

For shared lineages, the prevalence differed significantly among the host species, ranging from 1% to 47% (Figure 2). The prevalence among resident birds was significantly higher than that in migratory (*t*_1_ = 3.21, *R*^2^ = 0.12, *p* = 0.002) but not passing birds (*t*_1_ = 1.22, *R*^2^ = 0.08, *p* = 0.24), while the prevalence in migratory and passing birds presented no significant differences (*t*_1_ = 1.12, *R*^2^ = 0.05, *p* = 0.27).

To test whether closely related hosts are more likely to share lineages, we calculated the phylogenetic signal for the shared lineages (Table 1). A strong phylogenetic signal was detected in only one lineage, EMGOD06, which was detected mainly in tits. Apart from this, five lineages presented weak phylogenetic signals in prevalence, indicating that closely related host species presented a slightly higher similarity than those randomly selected from phylogeny. The remaining lineages showed no phylogenetic signal in prevalence, i.e., closely related host species did not present more similar rates of prevalence than the randomly selected species. 

### 2.3. Parasite Assemblages in the Different Host Groups

From 18 out of the 30 bird species, more than five samples were collected. In all three host groups, the prevalence of the shared lineages was significantly higher than that of other lineages (resident birds: *t*_1_ = 3.15, *R*^2^ = 0.14, *p* = 0.002; migratory birds: *t*_1_ = 3.45, *R*^2^ = 0.59, *p* < 0.001; and passing birds: *t*_1_ = 4.65, *R*^2^ = 0.08, *p* < 0.001).

**Figure 3:**
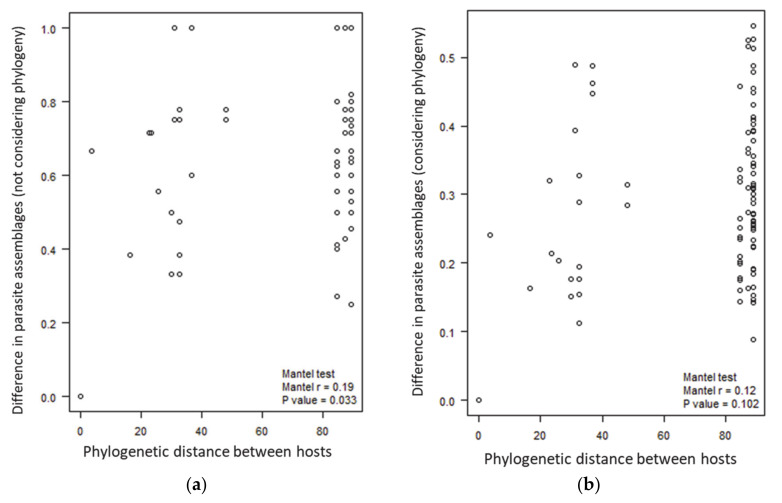
Mantel test between the phylogenetic distance of host species and the difference in the parasite assemblages harbored by each host: (**a**) not considering parasite phylogeny; (**b**) with parasite phylogeny considered.

To investigate the role of host phylogeny in shaping host–parasite associations, we tested the level of similarity in the parasite assemblages among the host species (Figure 3). A significant positive correlation was detected between the phylogenetic distance among these host species and the difference in the parasite assemblages (Mantel test, r = 0.19, *p* = 0.033); i.e., closely related bird species harbored more similar parasite assemblages than randomly chosen species. However, when the parasite phylogeny was taken into consideration (in terms of Faith’s phylogenetic β-diversity), the correlation was not significant (Mantel test, r = 0.12, *p* = 0.102).

## 3. Discussion

In this study, we investigated the assemblages of haemosporidian parasites in a wild bird community to detect whether bird species with different migration statuses share parasite lineages in multi-host communities and to consider the role of host phylogeny in shaping host–parasite associations. Our results showed that resident birds are more likely to share parasites with migratory birds breeding in the study site rather than passing birds, and such parasite sharing was not restricted by host phylogeny.

Although slightly more lineages were detected in the migratory birds than in the resident birds, the diversity of lineages did not differ greatly between birds with different migration statuses. Lineages detected in the different groups of birds were clustered together on the phylogenetic tree, suggesting that the migration status of the host was not the main driver of parasite diversification in this community. Migratory birds may accumulate infections during migration, but their associations with haemosporidian parasites are relatively loose when compared with resident birds. More than 70% of the lineages (69 out of 98) in the community were detected in the migratory or passing birds, but only 13 were also detected in the resident birds, indicating that the rest of the detected lineages may not be locally transmitted. In other words, most lineages infecting migratory birds and passing birds were acquired during their migration pathways and, apparently, were not transmitted in their breeding ground or stopover sites. As the associations between vectors and haemosporidian parasites are poorly studied, we do not know whether this is due to the lack of vectors [3] or because local birds are resistant to those lineages [21].

Lineages only detected in the migratory birds all presented a low prevalence, which may be the result of two processes: either the physiology of the birds during migration cleared the parasites in their bloodstream [18], or heavily infected birds could not survive migration [22]. During the annual migration, oxidative stress in the birds increases, and previous studies have shown that high-level oxidative stress in hosts can cause the death of *Plasmodium* parasites [23]. Therefore, it is possible that the infection intensity in host individuals is reduced during migration, and if this intensity is lower than the detection threshold, a low prevalence will be observed [24]. This assumption is also supported by the finding that bird species with longer migration distances present slightly lower levels of parasite diversity. On the other hand, during migration, hosts are assumed to have a relatively poor body condition [25], and those harboring heavy infections cannot finish the journey as a ‘cost of migration’, resulting in a relatively low prevalence in the population arriving at breeding grounds [16].

For the lineages detected in the resident birds, two-thirds were absent in the migratory and passing birds. According to the Combes filter hypothesis [26], the ability of a parasite to infect a given host depends on their frequency of encountering and compatibility with each other. In our study, parasite lineages restricted to local resident birds may not have adapted to infect other birds. At the time of encounter, they may have been immediately cleared by the host’s immune system, or were unable survive long enough to complete their life cycle [27]. Meanwhile, lineages restricted to resident birds presented significantly lower rates of prevalence than those also detected in migratory birds, which means that these lineages are less likely to spill over to other hosts because of the low encounter frequency, if insect vectors have little or no preference for bird hosts [28]. 

The local resident birds shared more lineages with migratory birds than with passing birds. This result is consistent with previous studies, showing that haemosporidian infections in migratory birds mostly occur in breeding grounds rather than stopover sites [17,29]. One possible explanation for this is that the immune system of birds is suppressed during the breading season as a ‘reproduction-tradeoff’ [30], making them more susceptible to infections than they are at other times of the year [16]. Moreover, migratory birds stay for much longer periods in breeding grounds than stopover sites, indicating that they may have a greater chance of exchanging parasites with allopatric birds, i.e., local residents and other migratory birds.

The prevalence of shared lineages in the resident birds was significantly higher than that in the migratory birds, suggesting that these lineages are more likely to expand their host range from resident birds to migratory birds than vice versa. It is worth noting that most of the shared lineages appeared to be host generalists based on the records of the MalAvi database [6], showing weak phylogenetic signals (if any) in prevalence among different hosts, suggesting that host phylogeny was not the main barrier to parasite transmission.

Phylogenetically closely related hosts presented similar parasite assemblies, indicating that co-evolution plays a role in shaping host–parasite associations in this community. This is a reasonable conclusion, as closely related hosts are believed to have similar immune systems [31] and, therefore, a similar susceptibility to parasite infections. When parasite phylogeny was considered, the trend remained but was not significant, suggesting that parasites may have different strategies when adapting to different host species. Whether this is related to the host specificity of the parasites remains unknown, but future studies on infection intensities among different host species may help to settle this issue.

## 4. Materials and Methods

### 4.1. Sample Collection and Haemosporidian Identification

During the breeding seasons (from mid-May to early July) from 2016–2019, wild birds were captured using mist nets in the Xiaolongmen Forest Park (39°57′54″ N, 115°26′00″ E) in southern Beijing. Blood samples were collected from the brachial veins and stored in 75% ethanol until the DNA extraction. The sampled bird species were identified as summer migrants, passing birds, and local residents according to their migration status, migration distance, and the length of time each bird spent in the study site, which are listed in Table S1, following the worldwide bird migration data [32], and were further corrected by local ornithologists. 

The DNA extraction was conducted using TIANamp whole genomic DNA extraction kits (Tiangen, Beijing, China) following the manufacturer’s protocol. Haemosporidian infections were identified using a nested PCR protocol amplifying a segment of the mitochondrial *cytochrome b* gene (*cyt b*) of the parasites, as described by Hellgren et al. [33], and positive samples were determined by the presence of the target band through 2% agarose gel scanning. All samples were tested at least twice to detect mixed infections or false negatives. All positive samples were sequenced from both directions using a 3730XL automatic sequencer (ABI, Foster City, CA, USA).

The obtained sequences were trimmed and aligned in Geneious Prime v. 2021.1.1 (http://www.geneious.com/, accessed on 9 May 2022) and compared with those compiled in the MalAvi database, using the BLAST module for the taxonomic identification. Mixed infections that could not be identified manually were marked as ‘undefined’ and excluded from further analyses. Haplotypes with at least one base pair difference from the existing lineages were defined as novel.

### 4.2. Phylogenetic Analysis

A Bayesian tree based on all the identified lineages was constructed using the MrBayes v.3.2.6 [34] module, implemented in Geneious Prime v. 2021.1.1, with the GTR + I + G nucleotide substitution model, which was selected by jmodelTest v.2.1.7 based on the AICc model selection [35]. Four heated Markov chains were run simultaneously over 1 million generations and sampled every 200 generations. The first 10% of the trees were discarded as ‘burn-ins’ from the posterior distribution. The convergence of the runs was subsequently checked by confirming the ESS values for the likelihoods and the majority of the parameters, and the consensus phylogenetic tree was plotted using FigTree v1.4.3 and midpoint rooted (http://tree.bio.ed.ac.uk/software/figtree/, accessed on 20 June 2022). To visualize the host range of each lineage, a heatmap presenting the number of host species for the detected lineage was mapped onto the phylogenetic tree using the ‘phylo.heatmap’ function in the phytools R package [36] in R v. 3.6.2 [37]. 

To test whether closely related hosts were more likely to be infected by the same lineages, we calculated the phylogenetic signal in the prevalence of the shared lineages using Pagel’s λ [38] with the phytools R package [36]. A Pagel’s λ equal to one is consistent with a Brownian motion model of trait evolution, indicating that the inspected lineage presented more similar prevalence in the closely related hosts than those chosen randomly from the phylogeny, while zero indicated that the prevalence among closely related hosts was not more similar than that of the others.

The phylogenetic tree was obtained from birdtree.org [39] using the ‘Phylogeny subsets’ module, and the prevalence rates of the shared lineages (i.e., lineages detected in birds with different migration statuses) were visualized using the phytools R package [36].

### 4.3. Statistical Analysis

The prevalence of each lineage was calculated for the host species, with more than five individuals sampled. The differences in prevalence and lineage diversity (the number of lineages detected in each species) among species with different migration statuses were tested using the *t*-test, and the correlation between the migration distance and parasite diversity in each species was investigated using the linear model test. All statistical analyses were compiled in R v. 3.6.2 [37].

We further compared the differences in parasite assemblage among the different birds based on the PhyloSor index, an analogous phylogenetic β-diversity metric based on Faith’s phylogenetic β-diversity [40], calculated using the phylo.beta.pair function in the betapart R package [41]. The significance was obtained using the bootstrap method, in which a migration status (migrant, passing, or resident) was randomly assigned to each bird species and repeated 1000 times. The null hypothesis was that there were no significant parasite assemblage differences among the bird groups.

To estimate whether host phylogeny plays a role in shaping host–parasite associations, we assessed the correlation between the host phylogenetic distance and the differences in the parasite assemblages. The null hypothesis was that there was no linear trend between the host species in terms of the phylogenetic distance and parasite assemblage differences. Two Mantel tests were carried out in parallel, with and without consideration of the parasite phylogeny. Phylogenetic distances among the host species were calculated using the ‘cophenetic’ function in the ape R package [42], and the Faith’s phylogenetic β-diversity of the parasite assemblages was calculated using the betapart R package [41].

## 5. Conclusions

Migratory birds are considered to be important transmitters of parasites between different sites. In this study, we investigated the parasite assemblages in a wild bird community and their correlation with the migration status and phylogeny of the hosts. Parasite lineages with higher prevalence rates were likely to be transmitted between birds with different migration statuses but were not restricted by host phylogeny. Migratory birds were more likely to gain parasites from resident birds at their breeding ground than at stopover sites, probably due to the longer period spent in sympatry with local birds and suppressed immune systems during the breeding season. More than two-thirds of the detected parasite lineages were restricted to either the resident or migratory birds. The main factor that blocked their transmission remains unclear, but future studies on vector–parasite associations may help to settle this issue.

## Figures and Tables

**Table 1:** Phylogenetic signal of the rates of prevalence of lineages detected in more than one host group. Pagel’s λ ranges from 0 to 1. An λ value close to 1 refers to a strong phylogenetic signal (i.e., closely related host species exhibit a similar prevalence), while an λ value close to 0 refers to a weak phylogenetic signal.

Lineage	Genus	Host Group	Pagel’s λ	*p* Value
PARMIN02	*Haemoproteus*	Res + Mig	6.7 × 10^−5^	1
TURDUS2	*Haemoproteus*	Res + Mig	6.7 × 10^−5^	1
BT5	*Leucocytozoon*	Mig + Pass	6.7 × 10^−5^	1
PARVEN08	*Leucocytozoon*	Mig + Pass	6.7 × 10^−5^	1
EMEL03	*Leucocytozoon*	Res + Mig	6.7 × 10^−5^	1
EMSPO05	*Leucocytozoon*	Res + Mig	0.63	0.13
PARVEN03	*Leucocytozoon*	Res + Mig	0.57	0.09
TARCYA01	*Leucocytozoon*	Res + Mig	0.64	0.45
EMGOD06	*Leucocytozoon*	Res + Mig + Pass	1	0.05
HYBOR02	*Leucocytozoon*	Res + Mig + Pass	6.7 × 10^−5^	1
GALLUS02	*Plasmodium*	Res + Mig	6.7 × 10^−5^	1
PADOM02	*Plasmodium*	Res + Mig	0.43	0.61
SGS1	*Plasmodium*	Res + Mig	6.7 × 10^−5^	1
TSUB01	*Plasmodium*	Res + Mig	6.7 × 10^−5^	1
ALARV04	*Plasmodium*	Res + Mig + Pass	0.45	0.47

## Data Availability

All obtained *cyt b* sequences from novel lineages were uploaded to GenBank and the MalAvi database. The accession numbers are: OP358327–OP358424.

## Supplementary Materials

The following supporting information can be downloaded at: https://www.mdpi.com/article/10.3390/ijms231810277/s1.
